# XMRV infection in human diseases

**DOI:** 10.1186/1742-4690-8-S1-A238

**Published:** 2011-06-06

**Authors:** Otto Erlwein, Mark J Robinson, Steve Kaye, Myra O McClure, Marjorie M Walker, Anup Patel, Wun-Jae Kim, Mongkol Uiprasertkul, Ganesh Gopalakrishnan, Takahiro Kimura, Kikkeri Naresh

**Affiliations:** 1 Section of Infectious Diseases, Jefferiss Research Trust Laboratories, Imperial College London, London, W2 1PG, UK; 2 Department of Histopathology, Imperial College London, London, W2 1PG, UK; 3 Department of Urology, Imperial College Healthcare Trust, London, W2 1PG, UK; 4 Department of Urology, College of Medicine, Personalised Tumor Engineering Research Centre, Chungbuk National University, Chungbuk, 361-763, Korea; 5 Department of Pathology, Faculty of Medicine, Siriraj Hospital, Mahidol University, Bangkok, 10700, Thailand; 6 Vedanayagam Hospital, RS Puram, Coimbatore,-641002, India; 7 Department of Urology, Jikei University, School of Medicine 3-25-8, Nishi-Shinbashi, Minato-ku, Tokyo, Japan; 8 Centre for Pathology, Hammersmith Hospital Campus, Imperial College Healthcare NHS Trust, London, UK

## 

The novel gammaretrovirus xenotropic murine leukemia virus-related virus (XMRV) was identified in human prostate cancer tissue in 2006, confirmed in 2009 and later linked to a second human condition chronic fatigue syndrome, CFS. These investigations, all carried out in the US, have not been reproduced in Europe or in China.

We found no evidence for XMRV infection in CFS. Moreover, we failed to find evidence of XMRV infection in UK prostate cancer patients and in prostate cancer tissue taken from patients in India, Korea, Thailand and Japan, or in cancers other than that of the prostate.

Our UK CFS patients were consistently XMRV-free. We did, however, generate false-positive results from prostate cancer patient tissue, despite the fact that the no-template controls in our PCR were consistently negative and the PCR for murine mitochondrial DNA was often also negative.

Sources of this contamination will be discussed in our presentation.

